# Genetic association of mosaic loss of chromosome Y with prostate cancer in men of European and East Asian ancestries: a Mendelian randomization study

**DOI:** 10.3389/fragi.2023.1176451

**Published:** 2023-05-31

**Authors:** Takuro Kobayashi, Tsuyoshi Hachiya, Yoshihiro Ikehata, Shigeo Horie

**Affiliations:** ^1^ Department of Urology, Graduate School of Medicine, Juntendo University, Tokyo, Japan; ^2^ Department of Advanced Informatics for Genetic Diseases, Graduate School of Medicine, Juntendo University, Tokyo, Japan

**Keywords:** genomic instability, mosaic loss of chromosome Y, prostate cancer, ageing, Mendelian randomisation analysis

## Abstract

**Background:** Genomic instability is a significant hallmark of aging and has a major impact on aging biology. Mosaic loss of chromosome Y (mLOY) in blood cells is a common chromosomal abnormality in aging men and is considered an indicator of genomic instability. Previous studies have indicated a connection between mLOY and prostate cancer risk, but the causal relationship has not been fully established.

**Methods:** To determine the causal effect of mLOY on prostate cancer, we conducted a Mendelian Randomization (MR) study in two ancestral groups. We utilized 125 and 42 mLOY-associated variants as instrumental variables (IVs) in European and East Asian GWAS of prostate cancer, respectively. Summary-level data on prostate cancer was obtained from the PRACTICAL consortium (79,148 cases and 61,106 controls of European ancestry) and the Biobank Japan consortium (5,408 cases and 103,939 controls of East Asian ancestry). A single population was used to assess the causal relationship in East Asian ancestry. Our main method for obtaining MR results was inverse-variance weighted (IVW), and we conducted sensitivity analyses to confirm the robustness of our results. Finally, we combined the estimates from both sources using a fixed-effects meta-analysis.

**Results:** Our MR analysis using the IVW method showed that a one-unit increase in genetically predicted mLOY was associated with an increased risk of prostate cancer in the PRACTICAL consortium (OR = 1.09%, 95% CI: 1.05–1.13, *p* = 1.2 × 10^−5^), but not in the Biobank Japan consortium (OR = 1.13%, 95% CI: 0.88–1.45, *p* = 0.34). Sensitivity analyses robustly indicated increased odds ratios for prostate cancer with every one-unit increase in genetically predicted mLOY for the PRACTICAL consortium. Furthermore, mLOY was found to be associated with prostate cancer risk in a meta-analysis of both sources (OR = 1.09%, 95% CI: 1.05–1.13, *p* = 8.0 × 10^−6^).

**Conclusion:** Our MR study provides strong evidence that higher mLOY increases the risk of prostate cancer. Preventing mLOY may be a means of reducing the risk of developing prostate cancer.

## 1 Introduction

Aging is a complex process that is associated with a variety of physiological changes, known as hallmarks of aging (López-Otín et al., 2013). One of these hallmarks is the genomic instability, and mosaic loss of Y chromosome (mLOY) in leukocytes has attracted much attention as a biomarker of aging-related genomic instability (Thompson et al., 2019). mLOY is a commonly observed structural event of the loss of the entire Y chromosome in a subset of cells, while the remainder of cells retain a normal Y chromosome (Hachiya et al., 2021). The prevalence of mLOY in white blood cells increases with age (Zhou et al., 2016), is more common in hematopoietic organs of smokers (Dumanski et al., 2015), and is also associated with a condition called clonal hematopoiesis of indeterminate potential (CHIP) (Zink et al., 2017; Ljungstrom et al., 2022). mLOY is associated with age-related diseases such as Alzheimer disease, cardiovascular disease, and cancer (Forsberg et al., 2014; Dumanski et al., 2016; Noveski et al., 2016; Zhou et al., 2016; Loftfield et al., 2018; Loftfield et al., 2019; Barros et al., 2020; Sano et al., 2022). The association between mLOY and solid tumors varies in strength by cancer type, and prostate cancer is one of the cancers strongly associated with mLOY (Noveski et al., 2016; Zhou et al., 2016).

Prostate cancer is a significant health concern for men, being the second most common cancer and the fifth leading cause of cancer death worldwide (Ferlay et al., 2021) (Sung et al., 2021). Despite its high incidence, the causes and mechanisms of prostate cancer are not fully understood. Established risk factors for prostate cancer include advanced age, a family history of cancer, and genetic factors (Leitzmann and Rohrmann, 2012). Recent study has suggested that mLOY events were observed more frequently in prostate cancer patients than cancer-free controls (Noveski et al., 2016). However, whether the genomic instability in leukocytes detected as mLOY is a causal risk factor of prostate cancer has yet to be elucidated from existing observational studies.

Mendelian randomization is an epidemiological method that uses genetic variants as instrumental variables (IVs) to assess the potential causal association between an exposure and an outcome (Burgess et al., 2013; Davey Smith and Hemani, 2014). According to Mendel’s laws, genetic variants are randomly allocated during meiosis. By utilizing genetic variants as instrumental variables, MR can reduce the impact of confounding factors and eliminate reverse causations. MR research, in conjunction with large-scale genome-wide association studies (GWAS), has become a popular method in recent years for examining the causal relationship between complex exposures (Guo et al., 2016). GWAS studies are notable for their large sample sizes and abundance of single nucleotide polymorphisms (SNPs), making GWAS-based MR a desirable approach (Grover et al., 2017).

The aim of this study was to investigate whether mLOY is a causal risk factor of prostate cancer in men of European and East Asian ancestries based on Mendelian randomization analyses leveraging recent large-scale GWAS of mLOY (n = 205,011 for Europeans, and n = 95,380 for East Asians) and prostate cancer (79,148 cases and 61,106 controls for Europeans, and 5,408 cases and 103,939 controls for East Asians).

## 2 Materials and methods

### 2.1 Data source

To define IVs of genetic loci associated with mLOY levels (i.e., the proportion of cells lacking the Y chromosome), we utilized a large-scale GWAS of 205,011 men of European ancestry from the United Kingdom Biobank (Thompson et al., 2019). The United Kingdom Biobank is a large-scale biobank cohort that randomly recruited approximately 500,000 adults in the United Kingdom between 2006 and 2010. Demographic information including sex, race, ethnicity, and geographic distribution is available. Statistically independent signals were defined using clustering at a distance of 1 Mb across all imputed variants with *p* < 5 × 10^−8^, imputation quality score >0.5 and minor allele frequency (MAF) > 0.1%. Genome-wide significant lead variants that were correlated with each other due to long-range linkage disequilibrium (r^2^ > 0.05) were excluded from further consideration. In this study, a long-range phasing approach was adapted to estimate a dichotomous classification called PAR-LOY, which uses allele-specific genotyping intensities in the pseudo-autosomal region (PAR). The European GWAS reported 156 autosomal mLOY-associated loci. We retrieved the association statistics (i.e., reference allele, alternate allele, beta coefficient, and standard error of beta coefficient) for the lead variants of the 156 loci.

Similarly, we obtained the association statistics from the East Asian GWAS of mLOY levels, incorporating 95,380 men of Japanese ancestry from the Biobank Japan (BBJ) (Terao et al., 2019). Biobank Japan is a hospital-based biobank that recruited approximately 200,000 Japanese individuals aged 20–69 years between 2003 and 2008. The GWAS analysis in BBJ tested 9,591,901 variants with r^2^ > 0.3 and MAF>0.005 for association with mLOY using Bayesian mixed model with bolt-lmm. Age, array, smoking, and disease status (prevalence >0.5% in subjects) were included as covariates. *p* < 5.0 × 10^−8^ was set as the genome-wide significant level. In this study, logarithm of R ratio (LRR) probe intensity data across multiple Y chromosome variants for each male subject was obtained, and mean LRR (mLRR-Y) was used as a proxy for mean Y chromosome dosage in circulating blood cells (Wright et al., 2017). The East Asian GWAS reported 50 independent variants in 46 loci associated with mLOY levels.

Regarding the outcome, we used a large-scale GWAS of prostate cancer involving 79,148 cases and 61,106 controls of European ancestry from the Prostate Cancer Association Group to Investigate Cancer Associated Alterations in the Genome (PRACTICAL) Consortium (Schumacher et al., 2018). PRACTICAL is a consortium of multiple case-control studies that recruited a total of over 80,000 prostate cancer cases and controls from Europe, North America, and Australia. The genome-wide association statistics of the European ancestry GWAS were downloaded from the GWAS Catalog (GWAS Catalog ID, GCST006085) (Sollis et al., 2023). The mean age of prostate cancer patients in PRACTICAL was 66 years, and the stage was low aggressive ((T0 or T1) and Gleason Score≤6 and PSA<10) in 12.1%, intermediate aggressive (T2 or Gleason Score = 7 or PSA between 10 and 20) in 37.9%, high aggressive ((T3,T4) or (N1) or (M1) or Gleason Score≥8 or PSA>20) in 26.8%, and advanced (defined as Gleason Score 8+, metastatic disease, PSA >100, or death from prostate cancer based on previous definition of aggressiveness (iCOGS) and available phenotype information) in 20.1%. After excluding low call rates (<95%) and high or low heterozygosity (*p* < 1.0 × 10^−5^), poorly imputed SNPs (r^2^ < 0.3), 201,598 SNPs remained (Davies et al., 2015). The effect estimate of each SNP was obtained using a fixed-effects meta-analysis combining the summary statistics from the OncoArray analysis and seven previous prostate cancer GWAS or high-density SNP panels of European ancestry imputed to the 1000 Genomes Project.

Of the 156 mLOY-associated variants identified by the European ancestry GWAS, 29 were not included in the genome-wide association statistics of the European ancestry GWAS of prostate cancer, and two ambiguous and palindromic SNPs (minor allele frequency >0.42) were excluded. Thus, we extracted the association statistics for the remaining 125 mLOY-associated variants, which were used as IVs for subsequent MR analysis of European ancestry (Supplementary Tables S1, S2).

The genome-wide association statistics of the East Asian GWAS was downloaded from the RIKEN Jenger (http://jenger.riken.jp/en/). The mean age of prostate cancer patients in the Biobank Japan cohort was 72.5 years, and the percentages of patients with PSA levels <4.0 U/mL, 4.0–9.0 U/mL, and ≥10.0 U/mL were 67.6%, 13.2%, and 15.9%, respectively (Ukawa et al., 2017). The percentages of patients with stage I, II, III, and IV disease were 0.0%, 24.4%, 7.3%, and 4.4%, respectively. In this study, samples were genotyped using either the Illumina HumanOmniExpressExome BeadChip or a combination of the Illumina HumanOmniExpress and HumanExome BeadChips. To ensure sample quality, all samples with a call rate of <0.98 or identified as outliers from East Asian clusters by principal component analysis using the genotyped samples and the three major reference populations (Africans, Europeans, and East Asians) in the International HapMap Project were excluded. For quality control of genotypes, variants were excluded if they met any of the following criteria: 1) call rate was <99%; 2) Hardy-Weinberg equilibrium (HWE) *p*-value was <1.0 × 10^−6^; and 3) there were fewer than five heterozygotes. After imputation, variants with an imputation quality of R^2^ < 0.7 were also excluded. A GWAS was performed using a GLMM called SAIGE, which includes a null logistic mixed model with genotype data and a leave-one-chromosome-out approach. Age and the top five principal components were included as covariates. The genome-wide significance level was set at a *p*-value < 9.58 × 10^−9^.

Of the 46 mLOY-associated variants identified by the East Asian GWAS, four variants were not included in the genome-wide association statistics of the East Asian GWAS of prostate cancer. We retrieved the association statistics for the remaining 42 variants (Supplementary Tables S3, S4).

In addition, summary data on smoking were obtained for sensitivity analysis; genome-wide association statistics for the 518,633 European ancestry GWAS on smoking were downloaded from the GWAS Catalog (GWAS Catalog ID, GCST007327) (Karlsson Linner et al., 2019). GWAS results were meta-analyzed from the United Kingdom biobank and from the Tobacco and Genetics (TAG) Consortium, and only SNPs with MAF >0.001 were analyzed. For the East Asian GWAS on smoking, we used the GWAS in BBJ of 67,773 case and 21,905 control individuals based on Japanese ancestry (Matoba et al., 2019). The study used quality control measures to ensure that only high quality genetic variants with MAF ≥0.5% and INFO score ≥0.7 were included in the analysis.

### 2.2 Mendelian randomization analysis

First, we performed MR analysis separately for Europeans and East Asians. We used the multiplicative random-effects inverse-variance weighted (IVW) method as the primary MR analysis (Burgess et al., 2013). This is a classical method of MR analysis, which estimates IVW meta-analyses of Wald ratio for each SNP on the outcome and provides an accurate estimate of the causal effect, when all SNPs are valid instrumental variables (Burgess et al., 2017). As a secondary analysis, WM states that the more SNPs are used as instrumental variables, the more reliable the causal estimates are. WM estimates can draw robust and correct conclusions, even if 50% of the weight comes from invalid IVs (Bowden et al., 2016).

To assess the robustness of our results, several statistical tests were also performed for heterogeneity. Cochran’s Q statistic for IVW was calculated to assess the heterogeneity between different SNPs (Bowden et al., 2015; Greco et al., 2015). Furthermore, MR-Steiger test was used to explore the possibility of reverse causality of prostate cancer for mLOY (Hemani et al., 2017). We also used several other sensitivity analyses, namely, MR-Egger (Burgess and Thompson, 2017), contamination mixture (ConMix) (Burgess et al., 2020), leave-one-out (Corbin et al., 2016), MR-PRESSO (Verbanck et al., 2018), and steiger filtering (Hemani et al., 2017).

As a further analysis, we aimed to exclude the influence of SNPs associated with smoking on the causal relationship between mLOY and prostate cancer. To achieve this, we conducted MR analysis after excluding SNPs whose directionality was stronger than that of mLOY, using the steiger filtering test. We also excluded SNPs significantly associated with smoking after the Bonferroni multiple testing correction, and then repeated the MR analyses to assess the causal relationship between mLOY and prostate cancer.

In a previous study, comparison of genetic architecture of mLOY between Europeans and East Asians revealed a strong genetic overlap in association with mLOY between the two populations (Terao et al., 2019). Therefore, we performed a meta-analysis of the two populations for the causal association between mLOY and prostate cancer. The ancestry-specific MR estimates were meta-analyzed using the fixed effect model (DerSimonian and Laird, 1986). *I* ^*2*^ statistic was used to assess heterogeneity between ancestries (Higgins and Thompson, 2002). *I* ^*2*^ values of <25%, 25%–75%, and >75% were defined as low, moderate, and high level of heterogeneity, respectively.

All statistical analyses were performed using the R language, version 4.1.2 (R Foundation for Statistical Computing, Vienna, Austria) with “TwoSampleMR” (version 0.5.6), “MendelianRandomisation” (version 0.7.0) and “metafor” (version 3.4–0) packages. Two-sided *p* < 0.05 was considered statistically significant. We reported all results with corresponding 95% confidence intervals.

## 3 Results

### 3.1 Genetic association of mLOY with prostate cancer in men of European ancestry

**FIGURE 1:**
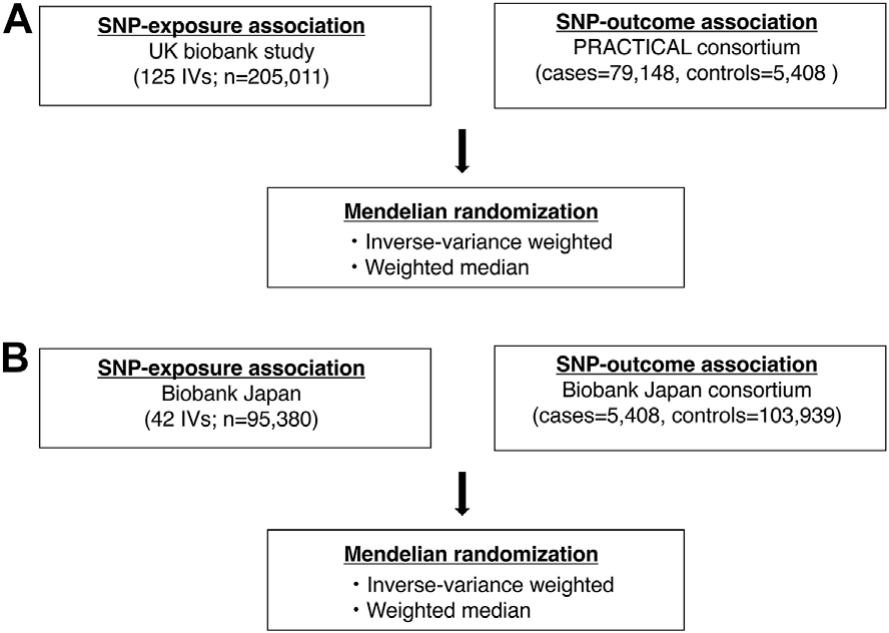
Analysis flowchart in this study. **(A)** shows a flowchart of the Mendelian randomization analysis in the European ancestry. **(B)** shows a flowchart of the Mendelian randomization analysis in the East Asian ancestry. SNP, single nucleotide polymorphism.

We conducted MR analysis to explore the causal relationship between mLOY and prostate cancer (Figure 1). The 125 mLOY-associated variants, which were identified by the European ancestry GWAS of mLOY levels and were included in the European ancestry GWAS of prostate cancer, were used as IVs for MR analysis (Supplementary Table S2).

**FIGURE 2:**
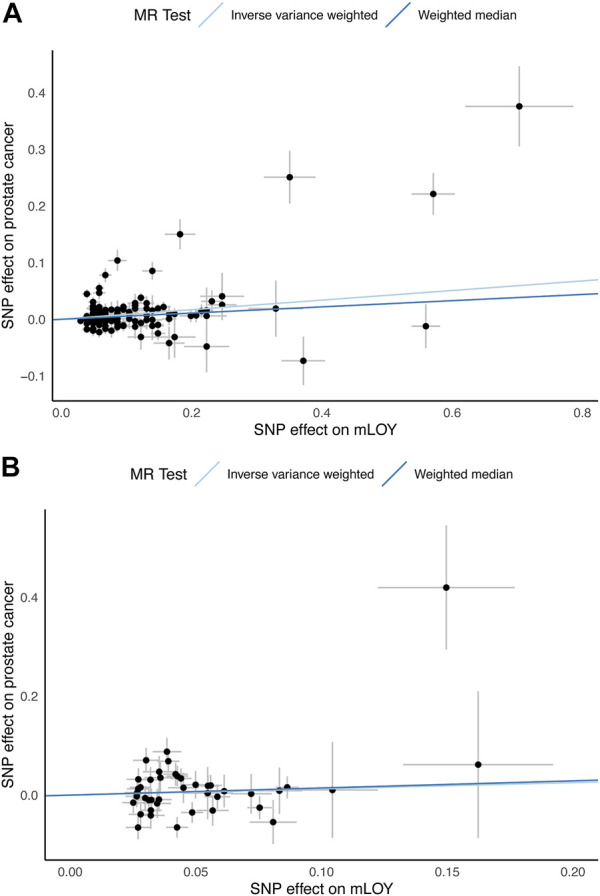
Scatter plots for MR analyses of the causal effect of mLOY on prostate cancer. **(A)** shows European ancestry data using PRACTICAL consortium. **(B)** shows East Asian ancestry data using Biobank Japan consortium. mLOY, mosaic loss of chromosome Y; MR, Mendelian randomization; SNP, single nucleotide polymorphism.

The primary analysis based on the IVW method suggests that genetically increased mLOY levels were associated with an increased risk of prostate cancer (OR, 1.09 [95% CI, 1.05–1.13]; *p* = 1.2 × 10^−5^) (Table 1; Figure 2). The secondary analysis using the WM method supported the finding from the primary analysis (OR, 1.06 [95% CI, 1.02–1.09]; *p* = 0.0017). Additionally, we used the MR-Steiger test to confirm that the direction of the effect was robust and that the instruments for mLOY affected prostate cancer susceptibility, not the reverse (*p* = 0).

**TABLE 1:** Prostate cancer Mendelian randomization analyses using mLOY SNPs as instrument variables.

Consortium	OR per unit mLOY (IVW method)	OR per unit mLOY (WM method)	*I* ^*2*^ *, P*_heterogeneity_	MR-steiger
PRACTICAL	1.09 (1.05–1.13), *p* = 1.2 × 10^−5^	1.06 (1.02–1.09), *p* = 0.0017	72.0%, *p* = 2.1 × 10^−37^	TRUE, *p* < 0.001
Biobank Japan	1.13 (0.88–1.47), *p* = 0.34	1.15 (0.87–1.54), *p* = 0.33	54.9%, *p* = 1.2 × 10^−4^	TRUE, *p* < 0.001

IVW, inverse variance weighted; OR, odds ratio; mLOY, mosaic loss of chromosome Y; MR, mendelian randomization; SNPs, single nucleotide polymorphisms; WM, weighted median.

### 3.2 Genetic association of mLOY with prostate cancer in men of East Asian ancestry

The 42 mLOY-associated variants, which were identified by the East Asian GWAS of mLOY levels and were included in the East Asian GWAS of prostate cancer, were used as IVs for MR analysis (Supplementary Table S4). Genetically predicted mLOY levels were not significantly associated with prostate cancer in the primary (OR, 1.13 [95% CI, 0.88–1.45]; *p* = 0.34) and secondary (OR, 1.15 [95% CI, 0.87–1.54]; *p* = 0.33) analyses (Table 1; Figure 2).

### 3.3 Meta-analyses between mLOY and risk of prostate cancer

**FIGURE 3:**
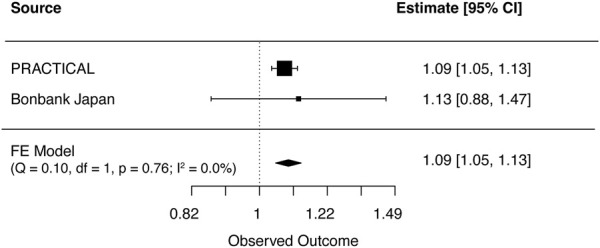
Meta-analysis results for genetically predicted mLOY in relation to prostate cancer. The horizontal line indicates 95% CI by using the IVW approach, squares indicate estimates, square size is proportional to sample size, and rhombus indicates meta-analytically pooled estimates’ 95% CI. df, degrees of freedom, FE, Fixed effect; IVW, inverse variance weighted; MR, Mendelian randomization; CI, confidence interval.

Cross ancestry meta-analysis of MR estimates showed that genetically predicted mLOY levels were associated with an increased risk of prostate cancer both in primary (OR, 1.09 [95% CI, 1.05–1.13]; *p* = 8.0 × 10^−6^) and secondary (OR, 1.06 [95% CI, 1.02–1.10]; *p* = 0.001) (Figure 3; Supplementary Figure S1). There was no heterogeneity in the MR estimates across ancestries in primary (Cochran’s Q statistics *p* = 0.76, *I* ^2^ = 0.0%) and secondary (Cochran’s Q statistics *p* = 0.55; *I* ^2^ = 0.0%) analyses.

### 3.4 Sensitive analysis

Additional analyses were performed to support these findings using different methods such as MR-egger and ConMix analysis (Supplementary Table S2; Supplementary Tables S4, S5). In both MR-egger and ConMix methods, mLOY was significantly associated with prostate cancer of European ancestry (MR-egger: OR, 1.11 [95% CI, 1.04–1.20]; *p* = 3.9 × 10^−3^, ConMix: OR, 1.04 [95% CI, 1.00–1.08]; *p* = 4.4 × 10^−4^). MR-PRESSO showed horizontal pleiotropy (global test *p* < 0.001) and 11 out of 125 variants were determined to be outliers. mLOY also showed a significant causal effect on prostate cancer in the outlier-corrected estimate (OR, 1.05 [95% CI, 1.02–1.07]; *p* = 9.0 × 10^−4^). Leave-one-out sensitivity analysis showed that individual SNPs did not affect the overall estimates (Supplementary Figure S2). However, no significant associations were observed for individuals of East Asian ancestry. The MR-egger and ConMix methods showed positive but nonsignificant associations with ORs of 1.21 and 1.09, respectively. MR-PRESSO showed horizontal pleiotropy (global test *p* < 0.001), and two variants out of 42 were identified as outliers. Even in the outlier-corrected estimate, mLOY did not show a significant causal association with prostate cancer (OR, 1.16 [95% CI, 0.92–1.47]; *p* = 0.22). A meta-analysis of each MR analysis showed consistent positive associations and low heterogeneity between ancestries (*I* ^*2*^ = 0.0%).

Steiger filtering was then applied to confirm the directionality of the association between genetic variants and prostate cancer in individuals of European and East Asian ancestry (Supplementary Tables S2, S4, S6). Three SNPs (rs57026767, rs6731121, and rs11258419) were excluded by Steiger filtering in the European dataset, while no SNPs were excluded in the East Asian dataset. The results showed that there was a significant association between mLOY and prostate cancer in individuals of European ancestry using IVW, WM, MR-egger, ConMix, and MR-PRESSO methods, with ORs ranging from 1.04 to 1.13. The results in the East Asian dataset were not altered by this filtering. A meta-analysis showed consistent results with low heterogeneity between studies (*I* ^*2*^ = 0.0%).

As further sensitivity analyses, we repeated the MR analyses excluding smoking-associated SNPs from the genetic IVs (Supplementary Tables S2, S4, S7, and S8). The results showed significant associations between mLOY and prostate cancer in individuals of European ancestry using IVW, WM, MR-Egger, ConMix, and MR-PRESSO methods (Supplementary Tables S7, S8). However, in individuals of East Asian ancestry, the all MR methods showed no significant association (Supplementary Tables S7, S8). The meta-analysis consistently showed a positive association with low heterogeneity across studies (*I* ^*2*^ = 0.0%) (Supplementary Tables S7, S8).

## 4 Discussion

There are several observational studies about association between mLOY and prostate cancer, but it was not unclear whether mLOY was the causal risk factor of prostate cancer. Our study aimed to determine the causal relationship between mLOY and the risk of prostate cancer, based on Mendelian randomization analysis and subsequent meta-analysis of European and East Asian ancestry populations. Results for the causal relationship between mLOY and prostate cancer varied by ancestry, with no causal relationship found in East Asian ancestry and a causal relationship in European ancestry. Furthermore, in a meta-analysis, mLOY was also a causal factor in prostate cancer. Our study is the first report to show a potential causal relationship between mLOY and prostate cancer based on different ancestries, supported by extensive sensitivity analyses.

The association of mLOY with the presence of prostate cancer has been reported in several previous studies. In case-control studies using DNA collected from peripheral blood, the mean Y/X ratio was shown to be significantly lower in the cancer patient group than in the control group (Noveski et al., 2016; Asim et al., 2020). A cohort study showed that elderly men with LOY in their peripheral blood had a higher risk of all-cause mortality, including nonhematologic cancer death and prostate cancer (Forsberg et al., 2014). A prospective cohort study found that a greater proportion of prostate cancer patients were mLOY at least 1 year prior to diagnosis and even higher after diagnosis, compared to non-cancer patients (Zhou et al., 2016). A large study of cancer-naive individuals from the United Kingdom Biobank showed an association between mLOY and overall cancer, but not with the risk of future prostate cancer (Loftfield et al., 2019). Another MR study using the United Kingdom Biobank and the PRCTICAL consortium found a causal association between mLOY and prostate cancer using genetic risk score, which supports the findings of the present study (Thompson et al., 2019).

Genetic factors that increase the likelihood of mLOY may impact cancer risk, given the role of genome instability in cancer development. Genomic instability, including chromosome rearrangements and loss, plays a crucial role in the formation of some cancers. Cancer progression is a complex process linked to changes in the genomes of cancer cells, but the mutation of genes is typically a slow process due to the DNA monitoring and repair enzymes (Hanahan and Weinberg, 2000; Stratton et al., 2009). Nevertheless, cancers still occur frequently, partly due to the malfunction of components of the genomic systems, including the p53 tumor suppressor protein, which can lead to genomic instability and the generation of mutant cells with selective advantages (Levine, 1997; Lengauer et al., 1998). The molecular mechanisms underlying mLOY have not yet been fully understood, but recent genetic studies suggest that mLOY may arise in hematopoietic stem and progenitor cells (HSPCs) (Terao et al., 2019; Thompson et al., 2019). Most mLOY-associated genetic variants have been found to be located within or near cell cycle genes involved in DNA synthesis, mitosis, damage response and apoptosis (Zhou et al., 2016; Terao et al., 2019; Thompson et al., 2019; Grassmann et al., 2020). These variants appear to affect HSPCs rather than more differentiated white blood cells. The strong association of mLOY with clonal hematopoiesis found in a large-scale whole-genome sequencing study supports this idea (Zink et al., 2017; Guo et al., 2020).

This study has several limitations. Firstly, the bi-directional MR analysis was not possible due to the unavailability of publicly accessible summary statistics for the relevant genetic variants. Secondly, different mLOY detection methods (PAR-LOY and mLRR-Y) were used in the European and East Asian mLOY GWAS, respectively (Terao et al., 2019; Thompson et al., 2019). Although it was better to use the same detection method for consistency, applying the same detection method was not feasible because we employed the summary GWAS data from different studies (Terao et al., 2019; Thompson et al., 2019). Thirdly, the use of both one-sample and two-sample MR methods may limit the generalizability of the findings to other populations. The two-sample MR method is beneficial when there is a lack of information or a small sample size in one of the populations. In these cases, the method combines data from multiple populations to increase the statistical power of the analysis (Davies et al., 2018). This was the case for the United Kingdom Biobank and PRACTICAL data, where the sample size was sufficient to provide accurate estimates of the causal effect of mLOY on prostate cancer. On the other hand, the one-sample MR method is useful when there is a large sample size within a single population, and it enables a more in-depth examination of the effect of the exposure on the outcome within that population (Minelli et al., 2021). This was the case for the BBJ data, where the sample size was smaller compared to that of the United Kingdom Biobank and PRACTICAL data, but still adequate to provide accurate estimates of the causal effect of mLOY on prostate cancer in the Japanese population. These limitations hamper our ability to draw conclusions about the potential causal mechanisms between mLOY and prostate cancer, and highlight the importance of making summary statistics for genetic variants readily accessible for MR and other genetic association studies. Finally, the MR analysis relies on the assumptions of Mendel’s laws of inheritance and causality, which may not always hold true in real-world scenarios. This study relied on MR estimates, and a clearer understanding of the relationship between mLOY and prostate cancer could be obtained through replication of our findings using individual-level data.

In conclusion, our study provides evidence for the causal relationship between mLOY and the risk of prostate cancer. Further research is necessary to fully understand the mechanisms by which mLOY increases the risk of prostate cancer.

## Data Availability

The original contributions presented in the study are included in the article/Supplementary Material, further inquiries can be directed to the corresponding author.
