# Supplementary material for: Genetic association of mosaic loss of chromosome Y with prostate cancer in men of European and East Asian ancestries: a Mendelian randomization study
Source: Front Aging. 2023 May 31;4:1176451. doi: 10.3389/fragi.2023.1176451 (PMC10264619; doi:10.3389/fragi.2023.1176451)
Supplement: Supplementary file 2 [file Table1.DOCX]

Supplementary Material

Genetic association of mosaic loss of chromosome Y with prostate cancer in men of European and East Asian ancestries: A Mendelian randomization study

**Takuro Kobayashi, Tsuyoshi Hachiya, Yoshihiro Ikehata, Shigeo Horie***

*** Correspondence:** Shigeo Horie: shorie@juntendo.ac.jp

# Supplementary Figures and Tables

## Supplementary Figures


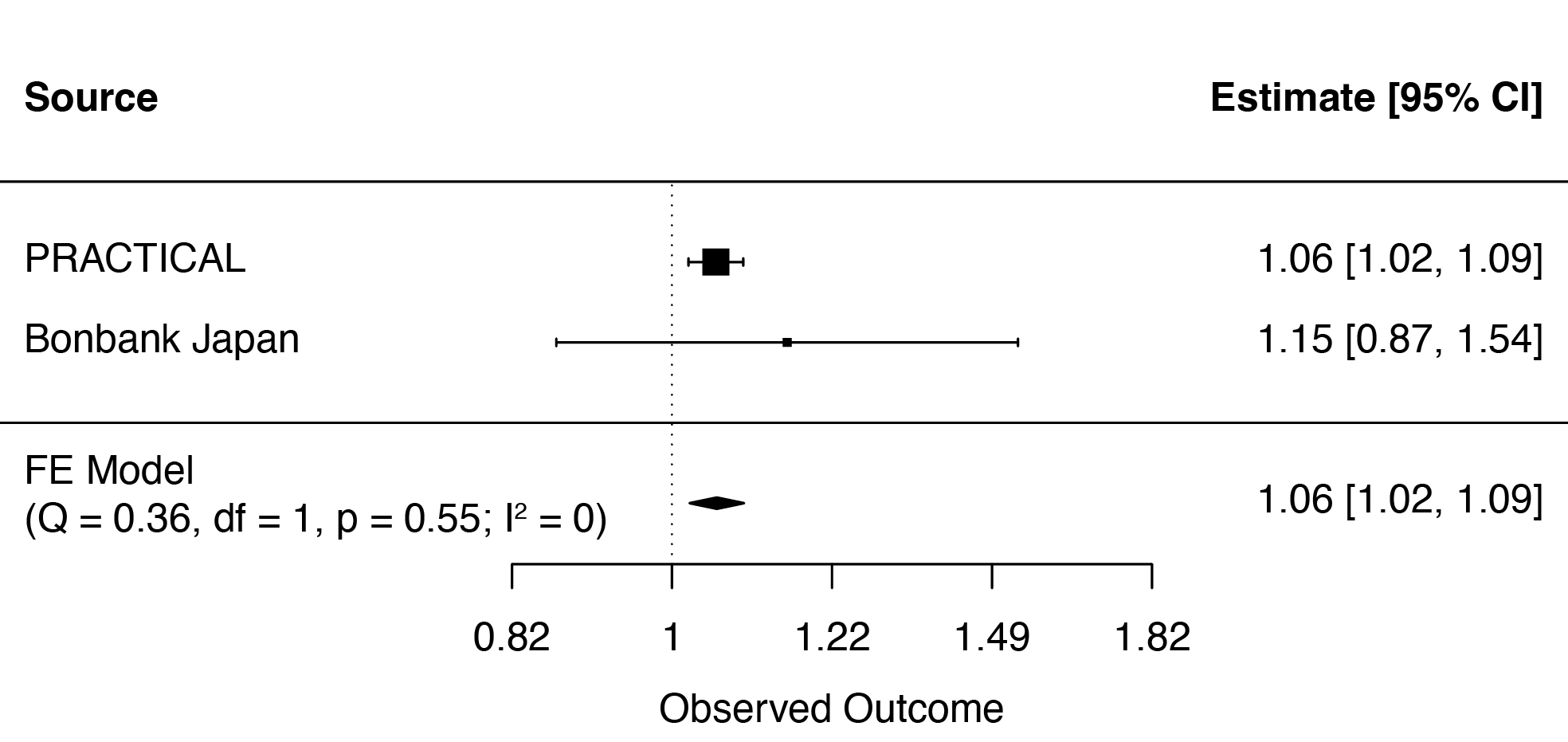


**Supplementary Figure 1.** Meta-analysis results for genetically predicted mLOY in relation to prostate cancer based on the WM method

The horizontal line indicates 95% CI by using the WM approach, squares indicate estimates, square size is proportional to sample size, and rhombus indicates meta-analytically pooled estimates’ 95% CI. df, degrees of freedom, FE, Fixed effect; WM, weighted median; MR, Mendelian randomization; CI, confidence interval.

**Supplementary Figure 2.** Leave-one-out sensitivity analysis using 125 mLOY-related SNPs as the exposure and prostate cancer as the outcome in European ancestry.

MR, Mendelian randomization

## Supplementary Tables

**Supplementary Table 1.** 156 mLOY-related variants identified by European ancestry GWAS at the mLOY levels.

**Supplementary Table 2.** 125 mLOY-related variants in the European Ancestral GWAS for prostate cancer and SNPs used in subsequent analyses.

**Supplementary Table 3.** 46 mLOY-related variants identified by East Asian ancestry GWAS at the mLOY levels.

**Supplementary Table 4.** mLOY-related variants in the East Asian Ancestral GWAS for prostate cancer and SNPs used in subsequent analyses.

**Supplementary Table 5.** MR sensitivity analysis of the causal relationship between mLOY and prostate cancer with 125 SNPs in the European ancestry and 42 SNPs in the East Asian ancestry.

**Supplementary Table 6.** MR sensitivity analysis and meta-analysis of the causal relationship between mLOY and prostate cancer with 122 SNPs in the European ancestry and 42 SNPs in the East Asian ancestry by Steiger filtering.

**Supplementary Table 7.** MR sensitivity analysis and meta-analysis of the causal relationship between mLOY and prostate cancer with 106 SNPs in the European ancestry and 36 SNPs in the East Asian ancestry after excluding smoking effects by Steiger filtering.

**Supplementary Table 8.** MR sensitivity analysis and meta-analysis of 103 SNPs in European ancestry and 36 SNPs in East Asian ancestry after excluding SNPs that significantly affect smoking.
